# The opportunities for and obstacles against prevention: the example of Germany in the areas of tobacco and alcohol

**DOI:** 10.1186/1471-2458-10-500

**Published:** 2010-08-19

**Authors:** Ulla Walter, Marc Suhrcke, Miriam G Gerlich, Till A Boluarte

**Affiliations:** 1 Hannover Medical School, Institute of Epidemiology, Social Medicine and Health System Research, Germany; 2 University of East Anglia, School of Medicine, Health Policy and Practice, Norwich, UK; 3 London School of Economics and Political Science, UK; 4 London School of Hygiene and Tropical Medicine, UK; 5 University of Witten/Herdecke, Germany

## Abstract

**Background:**

Recent years have seen a growing research and policy interest in prevention in many developed countries. However, the actual efforts and resources devoted to prevention appear to have lagged well behind the lip service paid to the topic.

**Discussion:**

We review the evidence on the considerable existing scope for health gains from prevention as well as for greater prevention policy efforts in Germany. We also discuss the barriers to "more and better" prevention and provide modest suggestions about how some of the obstacles could be overcome.

**Summary:**

In Germany, there are substantial health gains to be reaped from the implementation of evidence-based, cost-effective preventive interventions and policies. Barriers to more prevention include social, historical, political, legal and economic factors. While there is sufficient evidence to scale up prevention efforts in some public health domains in Germany, in general there is a comparative shortage of research on non-clinical preventive interventions. Some of the existing barriers in Germany are at least in principle amenable to change, provided sufficient political will exists. More research on prevention by itself is no panacea, but could help facilitate more policy action. In particular, there is an economic efficiency-based case for public funding and promotion of research on non-clinical preventive interventions, in Germany and beyond, to confront the peculiar challenges that set this research apart from its clinical counterpart.

## 1. Background

Recent years have seen a growing research and policy interest in primary prevention and health promotion in many European countries and beyond. Some of the interest has been fuelled by the expectation that prevention may mitigate at least a share of the expected, adverse fiscal consequences of demographic changes [1-3]. The evidence to support this specific expectation is at best mixed, however. Studies looking at various countries with different data for different health conditions find very inconsistent results about the cost-saving potential of prevention or of improved health in general [2,3]. Hence, while the hope for cost savings to be reaped from prevention remains highly questionable, there is no doubt that some preventive interventions and policies, even in the area of chronic diseases, can be a cost-effective investment bringing vital contributions to society's health and social welfare [4]. In this paper we take a closer look at the situation of prevention policy in Germany, within the European context. We confine the analysis to two areas of prevention that arguably serve particularly well to illustrate the potential for more and better prevention in Germany. We argue in the following section that Germany is a particularly interesting example in that the scope for prevention to improve health is significant, while at the same time - on the whole - efforts to engage more effectively in prevention have not been forthcoming, despite the available, if not abundant evidence on highly cost-effective preventive interventions. We apply the framework published by Kingdon [5] in order to assess possible obstacles for prevention policies in Germany. This framework focuses more on the flow and timing of policy action than on its component steps. Kingdon describes health policy change in a model of "three streams", a "problem stream" (Section 2.1), a "policy stream" (Section 2.2), and a "politics stream" (Section 2.3). He argues that only when these three streams are aligned and "flow" together does a window of opportunity for policy change open. However, if there are obstacles in any of these streams, substantial change will be hampered. In closing we point to the role and challenges of prevention research (Section 2.4) in this context. We focus on the risk factors of alcohol and tobacco, since these are well established in the "problem stream", and encouraging health policy changes in this field have in fact been implemented in some countries over the past years [6,7].

While this is a case study on Germany, a low priority on prevention is arguably a rather common phenomenon in many if not all European countries (and beyond). Hence, there is potential Europe-wide relevance in the present article.

## 2. Discussion

### 2.1 **The problem stream - the scope for prevention in Germany**

The rationale behind this stream is that a given situation has to be a) identified as a problem and b) amenable to human control, in order to have a chance at reaching the policy agenda. An assessment of comparative indicators, a chain of events, or other sources of feedback can indicate such an issue and the need of the government for some form of intervention.

Assessing the scope for health gains from more and/or "better" prevention in any given country is a challenging task. Here we make use of two (inevitably imperfect) proxy indicators for the extent of "preventive efforts" expended to date in Germany:

First, we review Germany's record in terms of two risk factors, alcohol use and smoking, that a) account for a large share of the burden of disease and b) for which there appears to be a widely shared consensus on the set of evidence-based preventive interventions that ought to be pursued [8,9]. Second, we look at Germany's implementation record of preventive policies or interventions. In both cases we use other European countries as benchmarks.

#### Burden of preventable disease

##### Smoking

In comparison with 14 other European countries, Germany shows, with 34% of its population above 15 years smoking on a daily basis, the second highest rate of daily smokers [10]. The daily smoking rate of 31% for women is the highest in the EU-15. While other data from the German national statistics office and available only for Germany shows lower absolute rates (due to differences in definitions and methods), these data allow us to examine trends over the recent decade: the percentage of smokers among respondents (aged 15-75) dropped from 28.3% in 1999 to 27.4% in 2003 and remained about the same in 2005 (27.2%), indicating only minor changes in smoking prevalence rates [11]. While smoking rates among young people (aged 12-25) dropped from 35.5% in 2004 to 32% in 2008 [11,12], current rates remain about as high as in other high-smoking countries in Europe, such as Austria and the Eastern European countries [11,13].

##### Alcohol

Germany ranks second among the EU-15 countries in terms of per capita alcohol consumption [10]. The average German consumes two litres more of pure alcohol per year than the average EU-25 citizen [14]. In particular the rise of excessive drinking patterns (especially binge drinking) among adolescents is alarming. About 75% of Germany's students aged 15-16 years consumed alcohol during the 30 days prior to a survey held in 2007, and 22% had been drunk during that period [13]. These figures are again well above the European (EU-25) average.

#### Prevention policies

##### Smoking

**Figure 1:**
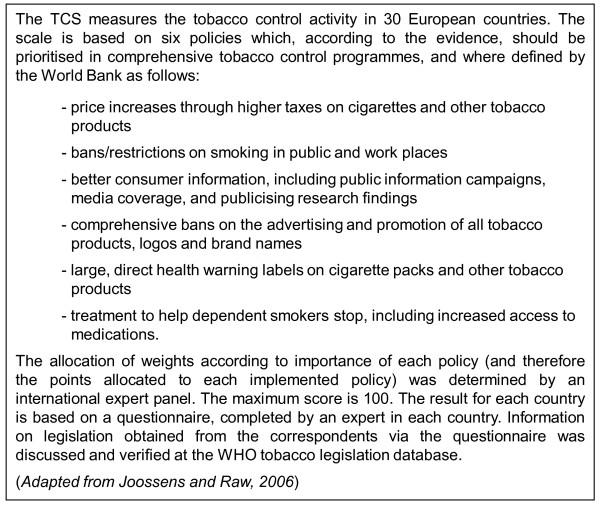
**The Tobacco Control Scale**.

The 2007 European Tobacco Control report confirms a low degree of anti-tobacco policy implementation in Germany [15]. The very few existing vending and advertising restrictions are largely based on voluntary agreements. Joossens and Raw [16] introduced the Tobacco Control Scale in 2005 to capture the extent of implementation of effective tobacco control policies, e.g., pricing, bans in public places, advertising bans, health warnings etc. [Figure 1]. In the latest ranking (2007), Germany came 27th among 30 European countries, trailed only by Greece, Luxembourg and Austria [7].

While some measures have been put in place to curb youth smoking (e.g., setting the official smoking age to 18 years, requiring a bank card to buy cigarettes from vending machines), overall efforts still appear half hearted and of limited success [17]. As of 2007, more than 80% of the adolescents reported access to tobacco as "very or fairly easy" [13], probably not least because of the comparative availability of vending machines: in 2008, there was one vending machine for about 205 citizens in Germany. Vending machines account for 13.5% of all tobacco sales in Germany [18]. About half the EU countries have banned sales from vending machines entirely [15].

##### Alcohol

A report [14] comparing the policy efforts in different European countries found that Germany (alongside Romania) had the lowest effective, purchasing power-adjusted tax rates on alcohol. At least partly due to low tax rates, the purchasing prices for alcohol in Germany are the lowest in the EU. In Germany alcohol can be sold virtually anywhere without a special sales licence. The minimum legal purchase age is 18 years for spirits and 16 years for other alcoholic beverages. However, a substantial percentage of adolescents reported having bought alcoholic beverages even if they were younger than the minimum age: 28.4% of under-16-year-old students reported having bought beer, while the numbers for wine or sparkling wine were 8.9%, for so-called "alcopops" 16.0% and for spirits 12.1% [19]. Advertisements, in particular for spirits, have been subject to some, if voluntary, regulation. Major sport events are often sponsored by manufacturers of alcoholic beverages (e.g., the 2006 World Soccer Cup and the Formula One Championship). Beside Ireland, Italy, Poland, the UK and Switzerland, Germany is one of the few countries that do not allow randomised breath testing (RBT) to identify and prevent drunk driving.

### 2.2 The policy stream - suitability of solutions for the German context

The objective of this stream is to describe the suitability of proposed solutions for a problem in terms of technical *feasibility *and *acceptability *within the population. In light of tight budget constraints, one of the major dimensions of feasibility nowadays is the efficiency (i.e., the value for money) of an intervention. The acceptability of an intervention has to be analysed within the context (i.e., social values, history) of the specific country in question. Therefore, we first scrutinise the cost-effectiveness of proposed solutions and then describe the contextual factors which might be obstacles to health policy change towards prevention.

#### The economic focus

Some might argue that part of the reason for the apparently low efforts on prevention in Germany (and elsewhere) might have to do with poor expected 'value for money' to be had from any preventive interventions. Such a view would, however, largely contradict current evidence, which suggests that in particular in the above-mentioned areas there are several highly cost-effective measures that could be implemented, and have been implemented in many other countries. In this section we first briefly review the cost-effectiveness evidence for interventions concerning the prevention of tobacco and alcohol use and subsequently extend the discussion to other areas, allowing us to also highlight the (no doubt existing) limitations in the evidence.

##### Smoking

There is substantial evidence on cost-effectiveness of policies to prevent and reduce tobacco consumption, even if much of this evidence comes from outside Germany [20]. In particular tobacco taxation has been identified as the most successful and cost-effective measure in smoking reduction [21,22]. The same studies also demonstrate the very favourable cost-effectiveness of banning smoking at work and in public places. For example a reduction of hospital admission for myocardial infarctions since introduction of smoke-free legislation was shown [23]. Greater uncertainty surrounds the (cost-)effectiveness of so-called "social marketing" approaches, i.e., the idea of achieving social health goals without repressive legislation, but rather by influencing attitudes by publishing information and lobbying for "healthful" behaviour through different marketing instruments [24]. Nevertheless, health warnings on cigarette packs seem to have reduced consumption by a detectable, yet small amount [21,22]. The analysis of mass media campaigns has so far produced mixed results. For instance, Abelson [25] claims that anti-smoking advertising and education has no detectable impact on aggregate consumption. However, anti-smoking campaigns may affect consumption indirectly by making tax increases and smoking ban policies more acceptable. Other studies undertaken in the UK mention more favourable results in terms of cost-effectiveness and conclude that mass media intervention would cost between £2,500 and £10,000 per Quality Adjusted Life Year (QALY) gained. These findings echo those from US-focused studies [26].

##### Alcohol

Several international systematic reviews have consistently shown alcohol taxation, drunk-driving interventions (e.g., a combination of a low blood alcohol limit and random breath testing) as well as regulations to reduce availability of alcohol through minimum legal purchase age to be highly effective in reducing alcohol-related harm. As a further example, brief interventions for drinkers at risk have been shown to be cost-effective [14,27]. In terms of cost-effectiveness, estimates suggest that an excise tax increase of about 50% is one of the most worthwhile measures, which would cost about $287 per Disability Adjusted Life Years (DALY) saved (excluding tax revenue), followed by a comprehensive advertising ban, which would cost $660 per DALY saved. Reduced retail access and RBT can also be considered cost-effective, at an estimated $1,208 and $2,741, respectively, per DALY saved [21].

The above interventions only address a share, if a substantial one, of the total preventable disease burden in Germany (and Europe), raising the question how much we know about cost-effectiveness of prevention in other areas. In recent years there has been a growing interest in such research, as documented by a series of major systematic reviews of the international literature [28-30]. While these studies do definitely document a significant amount of encouraging economic evidence in favour of primary prevention, they also acknowledge the limitations of that literature. For instance, on the basis of their systematic review of cost-effectiveness evidence for primary prevention of cardiovascular diseases (CVD) Schwappach and colleagues [30] found that

(1) Only very few studies assessed non-clinical broader health promotion interventions targeted at obesity, physical inactivity or dietary intake in children or young adults.

(2) Interventions targeting children or young people have only very rarely been evaluated in economic terms, despite the high level of expected benefits that are generally attributed to early prevention.

(3) The comparability of results between studies is severely limited by the marked differences in the methodologies and definitions applied.

From a German perspective, a further limitation is the scarcity of country-specific studies. In the review of Schwappach et al. mentioned above, a mere four studies (out of 195) related to Germany [30]. Similarly, in a systematic review of all economic evaluations carried out in the German health system context covering the period 1990-2004, only 36 out of 275 studies were found to consider preventive measures (excluding screening). Of those, only a small fraction was targeted at primary prevention or population-based approaches [30,31]. Hence, a rather strong case can be made for more research on the subject in Germany (and elsewhere).

While evidence is at best one out of many determinants of policy decisions, a more comprehensive evidence base could facilitate decision-making, which at present often follows more intuitive than scientific reasoning: the 2008 report of the compulsory health insurance funds [32] (one of the financial and organisational key players for prevention in the country) states that scientific evidence has been used to implement a preventive measure by the insurance funds in only 25% of the interventions initiated. Moreover, only 33% of the projects that were implemented by the insurance funds in a setting context used health parameters as evaluation criteria, and only 3% of the programmes have taken an economic perspective in their evaluation. By contrast, 71% of projects evaluated the satisfaction of the participants. Due to this lack of outcome research in the German context, several researchers try to translate evidence on preventive measures from other countries to the German healthcare system context. During such a process of translation it is important to consider structural conditions, which are embedded in cultural dimensions and historical developments. Every culture has its own meaning of community, patterns of decision-making, beliefs about health, help seeking behaviour, notions of individualism versus collectivism, attitudes toward the elderly, and approaches to problem-solving [33]. These structural conditions define a health system and affect an intervention's effectiveness, efficacy, costs, and efficiency. How to properly address these challenges in the translation process (e.g. through appropriate modelling or pragmatic clinical trials), is part of a persistent debate [34,35].

#### The focus of society

With seemingly few existing prevention efforts coupled with at least significant evidence on what could be done in the specific areas mentioned (and partly beyond), the question arises of why one observes this contrast. In this section we explore a set of not mutually exclusive hypotheses about obstacles to more and better (primary) prevention in Germany.

#### A preference for "individualism"?

Part of the explanation for a limited public policy response on prevention may be that the German population simply does not demand such policies as it runs against what some consider a very strong preference for individual freedom and a dislike of government interference in seemingly private business.

Interestingly, while such individual preferences are hard to quantify, public opinion surveys tend not to confirm a particularly individualistic preference among the German population. The World Value Survey asks respondents in many countries whether "people should take more responsibility to provide for themselves", or "the government should take more responsibility to ensure that everyone is provided for". Overall, Germany ranks among the European countries with the highest preference for government involvement [36]. There is, however, a clear distinction between Eastern and Western Germany in that the latter displays a significantly more individualistic attitude than the former, possibly due to different historical experiences [37].

In the debate on government's role in prevention, the overarching principle of individual freedom has frequently been invoked as a counter-argument to an augmented public policy stance on prevention, especially in relation to policies that would seek to change people's health-related behaviour. If the above opinion survey results are an accurate representation of the German population's preferences on government intervention in the health behaviour arena, then such counter-arguments would appear unfounded.

The introduction of non-smoking regulations in Germany (referred to in more detail below) may further illustrate this debate. Opponents to the regulations warn against what they see as an overly paternalistic approach that is at odds with other "free market" values [38]. However, several recent opinion polls have shown that legal regulations to ban smoking in public restaurants meets the approval of about three quarters of the population [39].

Hence, the individualism hypothesis is not really backed up by what the population as a whole feels, as opposed to a group of stakeholders and opinion leaders opposed to more intrusive prevention policies.

The question remains, however, how to better align individual preferences and public health goals in general. Research identified the concept of personal risk perception as an important factor for individual behaviour as well as for attitudes towards government intervention [40,41]. A lack of coherence between individual preferences and public health measures might arise, if individuals perceive restrictions in their personal freedom and choice as being disproportionate to the perceived risk of harm to the general public [41-43]. Two major findings are that first, people tend to evaluate risks of unwanted effects as higher for "others" than when applied to themselves, and second, people tend to be "myopic" towards risks that are delayed far into the future [41,44,45]. A possible pathway for public health policy might therefore be to strengthen messages of more short-term adverse events and externalities of behaviour, next to suitable solutions, in order to align the individual risk perception with community health goals. An example, referring to the case above, is the change in focus within the "smoking in public places" debate from the harm smoking does to the smoker herself to the harm inflicted upon third persons ("external costs") through passive smoking, which may have added momentum to smoking-related public health advocacy [46]. This idea could be transferred to the area of alcohol by articulating the effects of alcohol consumption on road traffic accidents and anti-social behaviour, as opposed to stressing "private" long-term outcomes such as liver cirrhosis. A further discussion of this mechanism and the role of public (health) policies in this task is beyond the scope of this article. However, more research in this area is needed.

#### History

Another potential obstacle to more prevention and health promotion arguably consists in their negative connotation with aspects of public health activities during the Third Reich era (1933-45), often defined by the term "Volksgesundheit" [47,48]. During this period the principle of "only a healthy nation is a strong nation" was taken as justification for, among others, interventions to enforce physical activity and to combat alcohol and tobacco use. The idea also incorporated the perverted ideas of racial hygiene and eugenics - including the notorious activities concerning "euthanasia" and mass sterilization of people with handicaps or deemed "unfit" [49]. This extremely negative historical legacy of prevention and public health may explain a degree of over-sensitivity even nowadays. Justified or not, the concern that public health (i.e., the health of the population as whole) might become more important than the individual's freedom and personal "pursuit of happiness" is often applied (more or less aggressively) during debates in Germany [50].

### 2.3 The politics stream - the German health political system: lack of incentives and dispersed power as obstacles for prevention

The politics stream describes the political context in which health policy is set. Since policy change is dependent on actors working together and reaching compromises, the attitudes and interests of each institution or individual involved needs to be aligned (e.g., through bargaining) in order to facilitate change. While some authors commonly use this section to describe changes in political actors over time (e.g., change in government), we apply this part of the framework to analyse the underlying structure of the German political system that is concerned with health policy. We try to identify whether structural problems in the organisation of legislation, the nature of social health insurance, or the influence of actors from private industry represent obstacles to the formulation and implementation of prevention policies.

#### The federal system and its actors

Germany's public policy in many areas, including healthcare, follows a federal (meaning shared sovereignty) rather than a centralised decision-making process. In particular, each state has the right to implement (or reject) laws on prevention. Taken together, there is a multitude of players, at national and state level, all contributing partially to prevention in Germany. The much-dispersed responsibilities and interests may well hinder the implementation of prevention policies that would otherwise benefit from coordination and harmonisation across regional borders. The recent debate around smoking ban laws is a case in point. In 2007 a national law was enacted banning smoking in public facilities, but the legislation that concerns smoking bans in restaurant and bars was transferred to the state level and resulted in a range of very diverse regulations, some of them complex, based on voluntary efforts with multiple exceptions for small bars, clubs and places holding traditional events [51]. Some innkeepers filed a lawsuit against the states' smoking prohibition at the Federal Constitutional Board. The court decided that such a law was in accordance with the German constitution because the government has the duty to safeguard the life and health of the general population. However, it has to be guaranteed that the federal legislation ensures both equality and consistency for all parties involved [52].

In response to the built-in exceptions, such as the freedom to run so-called "smoking clubs" to which only registered members have access, a petition for a referendum was initiated in the state of Bavaria (enrolment period November 19^th ^- December 2^nd^). The aim of the petition is a unified law for the protection of non-smokers *without *exceptions. The petition has exceeded the necessary proportion of 10% of eligible voters, so a referendum was scheduled for July 4^th ^2010 and approved by 61% of the population (voter participation 38%).

An additional, related problem may be that there is no central agency for public health in Germany that could help coordinate prevention policies where suitable. This leads to uncoordinated prevention measures on all federal levels (national, state, community), which tend to lack evaluation and/or evidence for effectiveness. In contrast, often programmes are continued, even though proved to be unsuccessful (e.g., certain behaviour-based approaches to prevent smoking in schools) [53].

In Germany, the Federal Centre for Health Education (BZgA) is a central institution for health promotion and preventive activities. Nevertheless, not all health topics are covered broadly. The BZgA focuses especially on national campaigns on tobacco and alcohol, as well as HIV prevention.

**Figure 2:**
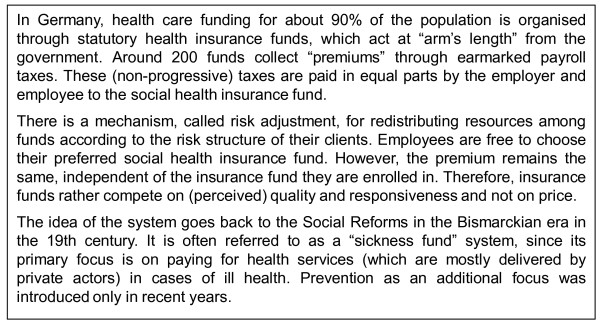
**A brief overview of the German Statutory Health Insurance system**.

Another coordination problem arises through the compulsory health insurance funds, which are at least in principle expected to play a major role in prevention [Figure 2]. There are approximately 200 funds of this kind in Germany, mostly organized at the state level. They have gained more legal rights recently to offer preventive measures to their customers. In 2008, insurance funds spent the highest amount ever for primary prevention: almost €340 million, which is equal to €4.83 per insurant, i.e., well above the requested amount of €2.74 for prevention per person and year. Substance abuse is not an explicit target of this programme, but is considered as one (minor) point within the scope of workplace health promotion and primary prevention activities within the "setting approach", e.g., measures in schools to improve life-skills [32]. The insurance funds are also allowed to incentivise their members through programmes such as monetary compensation or free fitness centre memberships. Critically though, they can only finance these incentives from cost savings. This is unlikely to be a sensible economic requirement, as programmes that would still provide good value (i.e., health gains) for money would have to be forgone, just because they do not meet the overly stringent criteria of cost-saving. Given that customers are free to switch to another insurance fund, these programmes are rather used as marketing tools than as concerted prevention efforts.

#### Particularly powerful industrial lobby

The overall lack of coordination and responsibility tends to produce a vacuum of interpretation sovereignty on prevention topics. This might be one reason why in Germany industrial lobbyists have a strong influence on legislation and fiscal measures, arguably particularly so in the health field. The severely watered down legislation of the smoking ban in public places may exemplify the influence of the tobacco lobby [54], as may the industry's strategies to oppose the advertisement ban at the European level as well as managing to maintain the establishment of different tax levels for different types of tobacco [39,55]. By contrast, some other preventive measures, e.g., vaccination for HPV, were introduced to the catalogue of publicly financed interventions at a surprisingly early stage (even before the major effectiveness studies were published), despite the explicit concerns by several research groups [56]. The decision process was accompanied by a large industry-funded media campaign.

### 2.4 The role of research

The case for greater public support for research on prevention, especially of the non-clinical sort, has already been highlighted above. Research on preventive interventions is subject to a market failure: the private actors do not have the incentive to engage in such research, because (a large share of) the resulting insights would become a public good that everybody could use, without having paid the often substantial research costs to arrive at that knowledge. From a sheer economic efficiency (and not even from any moral or public health) perspective, such knowledge is thus undersupplied compared to the social optimum. Hence, government ought to step in and help promote the production of evidence that the population at large would benefit from, but cannot produce if decisions are left to "the market" alone [57].

The development of a sound scientific basis and structured financial support can improve the impact of healthcare, as appears to have been the case in the German rehabilitation services sector. The establishment of a long-term research programme has improved the recognition and outcomes of rehabilitation services - a traditionally rather neglected area.

Prevention research, especially in the non-clinical arena, poses its own challenges, arguably the chief one being the attribution of changes in health outcomes to the intervention examined [58]. Population-based interventions tend to be more complex than the prescription and intake of a drug, and the (not flawless) gold standard of evaluation in the medical field - the randomised controlled trial (RCT) - cannot easily be implemented. Fortunately, though, there are other statistical methods that may allow the assessment of causality in such cases, but they have been underutilized in public health research to date [59,60].

A low public support for non-clinical research is of course not solely a feature of Germany but may well apply globally. This is exemplified by one of the rare attempts to quantify the extent of funding for health research in the UK recently, showing that prevention research accounts for only 2% of the overall research funding on health (with only a small fraction of this related more specifically to primary prevention or health promotion) [61]. While similar data do not exist for Germany, there is no reason to assume that the share is significantly higher in Germany. Nevertheless, there are also some positive trends: in 2003 the German Ministry of Education and Research made available more than € 20 million for research projects on primary prevention and health promotion. This programme is running from 2004 until 2012 and includes 60 research projects [http://www.knp-forschung.de/]. The Federal Centre for Health Education (BZgA) is a partner in this national comprehensive network to strengthen research in primary prevention, with the objective of establishing structures for sustainable primary prevention. Tasks include process definitions, evaluation of combined results and communication to political decision makers, research community and practical institutions. In the intermediation between science and the practical field, the BZgA could play an even greater role.

## 3. Summary

In Germany, substantial health gains could be reaped from the implementation of evidence-based, cost-effective preventive interventions and policies. However, applying the "three-streams" framework proposed by Kingdon [5], we showed that there are obstacles in the policy and politics streams that hinder substantial policy change. Barriers to more prevention include social, historical, political, legal and economic factors. While in the "problem stream" there is sufficient evidence to scale up prevention efforts in some public health areas in Germany, especially alcohol and tobacco, in general there is a comparative shortage of research on non-clinical preventive interventions. Some of the existing barriers in Germany are at least in principle amenable to change, provided the political will is sufficient. However, political decisions tend to be driven by short term election period perspectives, and hence the long term horizons of many prevention policies may not always align with political decision-makers' time horizons. More research on prevention by itself is no panacea but could help facilitate more policy action. In particular, there is an economic efficiency-based case for public funding and promotion of research on non-clinical preventive interventions [57], in Germany and beyond, to confront the peculiar challenges that set this research apart from its clinical counterpart.

It is beyond the scope of the present article to exhaustively discuss all the options for a more concerted effort on prevention in Germany. We highlighted only selected key points where improvement seems necessary and feasible. This does not readily provide us with a complete positive model of how prevention as a whole ought to be institutionalised in Germany or any other country. With wide variation in how prevention is delivered and institutionalised across European countries [62], a single *optimal *model has yet to emerge. While lack of the perfect model is no excuse for inaction on prevention in Germany, completing the picture of the features of such an ideal model of a national prevention policy should be high on the public health and social science research agenda. For this purpose international cross-country comparative research, rather than a single country case study would seem one promising approach.

## Conflicts of interests

The authors declare that they have no competing interests.

## Authors' contributions

UW and MS conceived the article and prepared the first draft. Both contributed equally to the paper. MGG helped further revise and finalise the draft. TAB contributed to the production of the first draft and subsequent revisions. All authors contributed to the drafting of the final version. All authors have read and approved the final manuscript.

## Pre-publication history

The pre-publication history for this paper can be accessed here:

http://www.biomedcentral.com/1471-2458/10/500/prepub
